# Nivolumab induced remitting seronegative symmetrical synovitis with pitting edema in a patient with melanoma: a case report

**DOI:** 10.1186/s13256-018-1579-1

**Published:** 2018-02-26

**Authors:** Linh Ngo, Eric Miller, Peter Valen, Elie Gertner

**Affiliations:** 1 University of Minnesota, Medicine Rheumatology Office, D615Mayo, 420 Delaware St SE, Minneapolis, MN 55455-034 USA; 2 Hennepin County Medical Center, Minneapolis, USA; 3 Minneapolis VA Medical Center, University of Minnesota, Minneapolis, USA; 4 Regions Hospital-Health Partners, St Paul, USA

**Keywords:** Nivolumab, RS3PE, Remitting seronegative symmetrical synovitis with pitting edema, Immune checkpoint inhibitor, Adverse effects

## Abstract

**Background:**

Novel immune checkpoint inhibitors have been often utilized for different types of malignancies as salvage therapy with varying success. One obstacle to immune checkpoint inhibitor use is the higher incidence of immune-mediated side effects that can prompt discontinuation of therapy. Remitting seronegative symmetrical synovitis with pitting edema has been described with immune checkpoint inhibitors only once previously. We report a case of a patient who developed remitting seronegative symmetrical synovitis with pitting edema related to immune checkpoint inhibitor therapy and stress that these symptoms can be managed without cessation of immune checkpoint inhibitor therapy.

**Case presentation:**

We present a 70-year-old white man who presented with 4 months of progressive inflammatory arthritis with pitting edema. He had been started on nivolumab therapy for his metastatic melanoma with excellent response prior to symptom onset. The symptoms started in his knees and subsequently involved both hands and feet. On evaluation, he was wheelchair bound and completely dependent for all activities of daily living. Evaluation revealed negative serological testing and plain film imaging. Ultrasound demonstrated diffuse flexor tenosynovitis and soft tissue swelling, and a diagnosis of remitting seronegative symmetrical synovitis with pitting edema was made. He was treated with orally administered corticosteroids (0.5 mg/kg per day) which improved his symptoms significantly and allowed him to regain prior independent functioning. His corticosteroids were tapered (0.15 mg/kg per day) but not discontinued and his nivolumab treatment was not interrupted. In follow up he continued to have stable control of his melanoma as well as his remitting seronegative symmetrical synovitis with pitting edema.

**Conclusions:**

In conclusion we present the first case of nivolumab-induced remitting seronegative symmetrical synovitis with pitting edema that is controlled by maintenance low-dose orally administered corticosteroids allowing for continuation of nivolumab therapy. Clinicians who encounter mild-to-moderate immune checkpoint inhibitor immune-mediated adverse effects can consider maintaining immune checkpoint inhibitor therapy with concomitant low-dose corticosteroids rather than abrupt cessation of the immune checkpoint inhibitor.

## Background

Immune checkpoint inhibitors can be used in the treatment of melanoma, non-small cell lung cancer, Hodgkin lymphoma, and small cell lung cancer. Often, they are used as salvage therapy. Nivolumab is a monoclonal IgG4 antibody that targets T-lymphocyte programmed cell death protein 1 (PD1) receptors and thereby disrupts regulation and promotes immune activation and anti-tumor response. Other checkpoint inhibitors, such as ipilimumab, target cytotoxic T-lymphocyte-associated antigen 4 (CTLA4). As a byproduct of immune activation, a spectrum of immune-related adverse effects including vitiligo, Sweet’s syndrome, mucositis, lymphocytic rash, non-infectious colitis, pneumonitis, hypophysitis, autoimmune thyroid disease, type 1 diabetes, Guillain–Barré syndrome, fulminant myocarditis, inflammatory arthritis, sicca syndrome, and uveitis have been extensively reported [1–12].

Remitting seronegative symmetrical synovitis with pitting edema (RS3PE) is a rare inflammatory arthritis characterized by acute onset symmetric distal tenosynovitis and pitting edema of hands and feet. It is usually responsive to low-dose glucocorticoids and occurs more often in older individuals with a male predominance [13]. Inflammatory markers may be elevated, but rheumatoid factor (RF) and anti-cyclic citrullinated peptides (CCP) antibodies are usually negative [14]. MRI and ultrasound findings reveal extensor tenosynovitis with lesser amounts of flexor tenosynovitis, joint synovitis, and subcutaneous tissue thickening [15]. RS3PE is a relatively poorly characterized syndrome that has been associated with malignancies, medications, and autoimmune disease. A review of the literature reveals a single previously reported case of nivolumab causing RS3PE [16]. In that case, nivolumab was held for resolution of the RS3PE and eventually discontinued due to malignancy progression. It is unclear if holding nivolumab contributed to malignancy progression or whether RS3PE would have progressed with continued nivolumab.

## Case presentation

A 70-year-old white man developed right knee pain and swelling followed by left ankle pain and swelling over a week. Over the next 4 months, his symptoms progressed to include both knees, both feet, and both hands. Due to the severity of his symptoms he was unable to ambulate or carry out normal activities of daily living. He initially took ibuprofen 800 mg three times daily with some mild improvement, but at the time of presentation, it offered no relief.

In addition, he endorsed morning stiffness that persisted for most of the day. Due to the stiffness in his joints, he could no longer ambulate and presented to our clinic in a wheelchair. He previously was fully functional and independent in his activities of daily living. He was an avid fisherman and was unable to pursue his interests at all.

### Medical history

His past medical history was significant for metastatic melanoma initially diagnosed 2 years ago. His initial lesion was located over the left side of his neck and he had a Mohs procedure with negative margins. He was monitored closely for 1.5 years until he was found to have new right lower lobe lung nodules on positron emission tomography (PET)/computed tomography (CT) with increased fluorodeoxyglucose (FDG) uptake. Wedge resection of the right lower lobe revealed metastatic melanoma with wild type BRAF and no C-KIT mutations. Continued surveillance demonstrated an increasing number of right pulmonary nodules over the next 6 months. Dual therapy nivolumab (1 mg/kg every 3 weeks for four doses followed by 240 mg every 2 weeks) and ipilimumab (3 mg/kg every 3 weeks) immunotherapy was started. After the second cycle of his immunotherapy he developed severe non-infectious colitis requiring hospitalization. His immunotherapy was stopped and his colitis resolved with supportive care and glucocorticoids. Without further immunotherapy, he developed new left pulmonary nodules within 6 months that were increasing in size. Single agent immunotherapy with nivolumab (240 mg every 2 weeks) was started 4 months before his presentation to Rheumatology. With single agent immunotherapy, the pulmonary nodules receded fully and no further metastatic disease was seen on subsequent PET/CT imaging 3 months later.

His medical history was also notable for hypertension and benign prostatic hypertrophy.

### Medications and allergies

He was treated with hydrochlorothiazide, aspirin, and nivolumab. He had no known drug allergies.

### Family and social history

There was no family history of connective tissue disease or inflammatory arthritis. His mother died from colon cancer in her 80s and his father had coronary artery disease. He was married with three living children. He served in the Navy during the Vietnam War and worked as a mechanic after his military service until retirement. He denied any history of recreational drug or alcohol use. He reported a 20-pack year tobacco smoking history, but quit over 10 years ago.

### Review of systems

He denied having any chest pain, shortness of breath, rashes, oral or nasal ulcers, alopecia, Raynaud’s disease, fevers, chills, night sweats, or unintended weight loss. He did endorse feeling weak because of his chronic condition.

### Physical examination

**Fig. 1:**
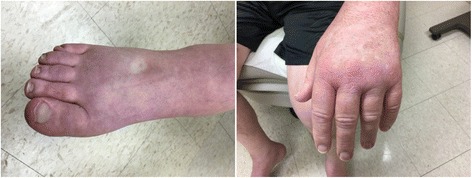
Soft tissue swelling and pitting edema of the hand and foot

He appeared his stated age and in no apparent distress. His temperature was 37 °C, blood pressure 116/78, heart rate 70 beats per minute, and oxygen saturation 100% on ambient air. His musculoskeletal examination was significant for tender boggy synovitis of his bilateral metacarpophalangeal joints (MCPs), proximal interphalangeal joints (PIPs), wrists, elbows, knees, ankles, and metatarsophalangeal joints (MTPs). There was no palpable effusion in any joint but he had significant soft tissue pitting edema present over his extremities. There was +3 pitting edema over the dorsum of both hands and feet extending up to his wrists and mid-shins respectively. There was mild erythema and warmth present over his joints, most notable over his MCPs (Fig. 1). There was decreased range of motion in his hands, feet, ankles, elbows, and knees. There also were extensor tendon rubs noted on range of motion of his MCPs bilaterally by palpation and auscultation. The remainder of the musculoskeletal examination and general physical examination was unremarkable. There were no rheumatoid nodules noted on examination.

### Laboratory evaluation

**Fig. 2:**
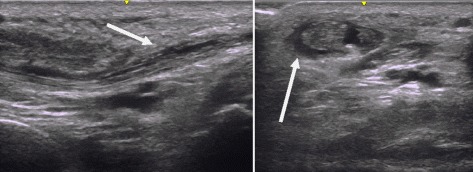
Longitudinal and transverse views of an extensor tendon of the wrist demonstrating tenovitis and tendinopathy (*arrows*)

**Fig. 3:**
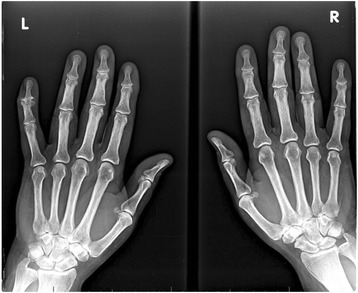
Plain films of both hands

Results of the laboratory evaluation are shown in Table 1. Our patient’s erythrocyte sedimentation rate and C-reactive protein (CRP) were quite elevated. The remainder of his laboratory tests was unremarkable. Ultrasound and X-ray imaging of his hands were obtained (Figs. 2 and 3) demonstrating soft tissue swelling and extensor tenosynovitis. There were no erosions present.

**Table 1:** Laboratory values

Laboratory test	Initial visit	Normal values
WBC count, × 10^9^/liter	3.8	4.0–11.0
Hemoglobin, gm/dl	12.2	11.5–15.7
Platelets, × 10^9^/liter	233	150–400
AST, units/liter	42	10–47
ALT, units/liter	36	7–45
LDH, units/liter	125	88–192
ESR, mm/hour	119	0–20
CRP level, mg/dl	113	< 5.0
Ferritin, ng/ml	672	25–345
TSH, mIU/L	1.73	0.4–5.0
Hepatitis C antibody	Negative	Negative
Hepatitis B surface antigen	Negative	Negative
Hepatitis B core antibody	Negative	Negative
Antinuclear antibodies	Negative	< 1:40
Rheumatoid factor	Negative	< 12
CCP	Negative	< 20

*ALT* alanine aminotransferase, *AST* aspartate aminotransferase, *CCP* cyclic citrullinated peptides, *CRP* C-reactive protein *ESR* erythrocyte sedimentation rate**,** *LDH* lactate dehydrogenase, *TSH* thyroid-stimulating hormone, *WBC* white blood cell

### Patient’s course

He was started on prednisone 40 mg (0.5 mg/kg per day) and tapered gradually over the course of 6 weeks to 10 mg daily. He had a very rapid response to the prednisone with almost complete resolution of his symptoms. Once his prednisone was decreased below 10 mg he began noticing a steady return of his symptoms. During this time period he continued treatment with nivolumab and on surveillance imaging he had complete resolution of metastatic disease. Due to the marked response of his melanoma to immunotherapy, it was felt that paraneoplastic RS3PE was unlikely. Although at the time there were no published reports of nivolumab or other checkpoint inhibitors causing a RS3PE picture, it was felt that because of the temporal relationship between the nivolumab and the acute onset of his symptoms that they were related. The numerous previously described autoimmune conditions associated with checkpoint inhibitors raised the possibility that this presentation of RS3PE was another rheumatological manifestation. The clinical dilemma we were left with was that our patient had previously demonstrated a very rapid relapse of his stage 4 melanoma when off treatment, yet was incapacitated with the side effect of the treatment. After a careful discussion with his oncologist, we elected to maintain a steady dose of prednisone of 7.5 mg daily to control rheumatological symptoms and continue nivolumab. At 9 months, he demonstrated minimal pitting edema, no inflammatory arthritis, and continued full response from nivolumab therapy.

## Discussion

RS3PE is a syndrome first described in 1985 as a distinct type of seronegative rheumatoid arthritis [17]. It is characterized by symmetrical distal pitting edema with synovitis of the hands and feet that is generally very responsive to low-dose glucocorticoids. It is reportedly more common among males age 60 and older [18, 19]. The incidence of RS3PE has been reported at 0.09% in a large Japanese cohort [20].

Multiple subtypes of RS3PE have been described, including an idiopathic form, as well as forms which may coexist with other autoimmune diseases such as Sjögren’s syndrome. It has also been reported as a paraneoplastic or medication-induced syndrome. Some studies suggested that idiopathic RS3PE falls in the spectrum of polymyalgia rheumatica (PMR) given the similar demographic characteristics and laboratory findings [21]. In a prospective study involving 23 patients with RS3PE syndrome, all patients were found to have hand and/or foot tenosynovitis on MRI. In the same study, patients with RS3PE were compared to patients with PMR and were found to have similar demographic, clinical, and MRI findings, suggesting both may be part of the same disease process. However, there are also distinctions between RS3PE and PMR. RS3PE has male predominance, generally requires a shorter duration and cumulative dose of glucocorticoid use, and has a lower frequency of symptoms and relapse. On the other hand, PMR is associated with a longer average duration of glucocorticoid use, female predominance, higher relapse rate, and RF positivity in up to 16.5% of patients [22, 23].

Paraneoplastic RS3PE presents in a similar fashion to idiopathic RS3PE but tends to have a more severe course with higher rates of recurrence [13]. In addition, patients with paraneoplastic RS3PE often display systemic symptoms and may not respond to glucocorticoids as well as those with idiopathic RS3PE. Often, RS3PE onset precedes the onset of malignancy [21, 23]. Overall rates of malignancy in patients with RS3PE have been estimated to be 20% when pooling data from European, American, and Japanese cohorts [23]. Paraneoplastic RS3PE is commonly associated with solid malignancies including prostate, gastric, and colon adenocarcinomas. However, it has been seen in hematologic malignancies as well, including non-Hodgkin lymphoma and chronic lymphoid leukemia [21]. Melanoma has not been reported to cause RS3PE.

Autoimmune disease-related and infection-associated RS3PE have been reported in small case series. Conditions including systemic lupus erythematosus, Sjögren’s syndrome, polyarteritis nodosa, and ankylosing spondylitis have been associated with RS3PE [24–27]. Infections including parvovirus and *Streptobacillus moniliformis* have been reported to cause RS3PE [28–30].

Medication-induced RS3PE is uncommon but drugs including insulin, rifampicin, and dipeptidyl peptidase-4 inhibitors have been associated with RS3PE [31–33]. Recently a case of an 80-year-old man with stage IV BRAF wild type melanoma treated with nivolumab who developed signs and symptoms consistent with RS3PE after the initiation of nivolumab was reported [16].

RS3PE has been associated with elevated levels of vascular endothelial growth factor (VEGF) in both paraneoplastic and connective tissue disease-related patients, which decrease with glucocorticoid use [34, 35]. VEGF is a potent vasodilator, increases vascular permeability, and plays a large role in angiogenesis [36]. As a result, VEGF contributes to a neoplasm’s ability to grow, invade, and metastasize [37]. Other biomarkers that maybe elevated in patients with RS3PE include matrix metalloproteinase 3 (MMP3) and interleukin-6 [38].

The cornerstone of treatment of RS3PE is treating any underlying disorder. Neoplastic RS3PE in particular may benefit from treatment of the underlying malignancy as those patients may not respond to glucocorticoids as well. In cases of idiopathic RS3PE, prognosis is favorable and anecdotal evidence suggests glucocorticoids are highly effective and can be tapered over several weeks to the lowest effective dose with the goal of maintaining remission for 12 to 18 months [39–42]. Our patient was in a difficult clinical scenario with advanced stage 4 melanoma and few options for therapy. His malignancy responded well to nivolumab and temporary cessation caused a relapse in his disease. Survival of patients with stage 4 melanoma is approximately 15 to 20% at 5 years in patients with metastasis to other parts of the skin and lymph nodes [43, 44]. In patients with metastases to other organs, survival is lower.

## Conclusions

Our case suggests that perhaps in some situations treating the symptoms with low-dose glucocorticoids can provide adequate checkpoint inhibitor side effect relief while still maintaining treatment for the underlying malignancy. This is a potentially desirable option in patients with metastatic malignancies in which no further therapy options are available.

## Abbreviations

CCP: Cyclic citrullinated peptides
CRP: C-reactive protein
CT: Computed tomography
CTLA4: Cytotoxic T-lymphocyte-associated antigen 4
FDG: Fluorodeoxyglucose
MCPs: Metacarpophalangeal joints
MMP3: Matrix metalloproteinase 3
MTPs: Metatarsophalangeal joints
PD1: Programmed cell death protein 1
PET: Positron emission tomography
PIPs: Proximal interphalangeal joints
PMR: Polymyalgia rheumatica
RF: Rheumatoid factor
RS3PE: Remitting seronegative symmetrical synovitis with pitting edema
VEGF: Vascular endothelial growth factor
